# Analysis of the Basidiomycete *Coprinopsis cinerea* Reveals Conservation of the Core Meiotic Expression Program over Half a Billion Years of Evolution

**DOI:** 10.1371/journal.pgen.1001135

**Published:** 2010-09-23

**Authors:** Claire Burns, Jason E. Stajich, Andreas Rechtsteiner, Lorna Casselton, Sean E. Hanlon, Sarah K. Wilke, Oleksandr P. Savytskyy, Allen C. Gathman, Walt W. Lilly, Jason D. Lieb, Miriam E. Zolan, Patricia J. Pukkila

**Affiliations:** 1 Department of Biology, Indiana University, Bloomington, Indiana, United States of America; 2 Plant Pathology and Microbiology, University of California Riverside, Riverside, California, United States of America; 3 Department of Biological Sciences, University of California Santa Cruz, Santa Cruz, California, United States of America; 4 Department of Plant Sciences, University of Oxford, Oxford, United Kingdom; 5 Department of Biology, University of North Carolina, Chapel Hill, North Carolina, United States of America; 6 Department of Biology, Southeast Missouri State University, Cape Girardeau, Missouri, United States of America; Fred Hutchinson Cancer Research Center, United States of America

## Abstract

*Coprinopsis cinerea* (also known as *Coprinus cinereus*) is a multicellular basidiomycete mushroom particularly suited to the study of meiosis due to its synchronous meiotic development and prolonged prophase. We examined the 15-hour meiotic transcriptional program of *C. cinerea*, encompassing time points prior to haploid nuclear fusion though tetrad formation, using a 70-mer oligonucleotide microarray. As with other organisms, a large proportion (∼20%) of genes are differentially regulated during this developmental process, with successive waves of transcription apparent in nine transcriptional clusters, including one enriched for meiotic functions. *C. cinerea* and the fungi *Saccharomyces cerevisiae* and *Schizosaccharomyces pombe* diverged ∼500–900 million years ago, permitting a comparison of transcriptional programs across a broad evolutionary time scale. Previous studies of *S. cerevisiae* and *S. pombe* compared genes that were induced upon entry into meiosis; inclusion of *C. cinerea* data indicates that meiotic genes are more conserved in their patterns of induction across species than genes not known to be meiotic. In addition, we found that meiotic genes are significantly more conserved in their transcript profiles than genes not known to be meiotic, which indicates a remarkable conservation of the meiotic process across evolutionarily distant organisms. Overall, meiotic function genes are more conserved in both induction and transcript profile than genes not known to be meiotic. However, of 50 meiotic function genes that were co-induced in all three species, 41 transcript profiles were well-correlated in at least two of the three species, but only a single gene (*rad50*) exhibited coordinated induction and well-correlated transcript profiles in all three species, indicating that co-induction does not necessarily predict correlated expression or vice versa. Differences may reflect differences in meiotic mechanisms or new roles for paralogs. Similarities in induction, transcript profiles, or both, should contribute to gene discovery for orthologs without currently characterized meiotic roles.

## Author Summary

Meiosis is the part of the sexual reproduction process in which the number of chromosomes in an organism is halved. This occurs in most plants, animals, and fungi; and many of the proteins involved are the same in the different organisms that have been studied. We wanted to ask whether the genes involved in the meiotic process are turned on and off at the same stages of meiosis in organisms that separated a long time ago. To do this we looked at three fungal species, *Saccharomyces cerevisiae* (baker's yeast), *Schizosaccharomyces pombe* (a very distantly related fungus of the same phylum), and *Coprinopsis cinerea* (a mushroom-forming fungus of a different phylum), which had a common ancestor 500–900 million years ago (in comparison, rats and mice separated ∼23 million years ago). We lined up meiotic stages and found that gene expression during the meiotic process was more conserved for meiotic genes than for non-meiotic genes, indicating ancient conservation of the meiotic process.

## Introduction

Meiosis is a specialized cell division process in which one round of DNA replication is followed by two divisions to produce haploid products. The basidiomycete mushroom *Coprinopsis cinerea* (also known as *Coprinus cinereus*) [1] is ideal for eukaryotic meiotic studies due to its short, well-defined life cycle and the highly synchronous development of both the mushroom and its meiotic tissues [2]. The mechanisms and molecular machinery associated with meiosis are well-conserved within eukaryotes, albeit with some modifications observed in widely studied systems, for example, the lack of synaptonemal complex (SC) in *Schizosaccharomyces pombe* (reviewed in [3], [4]), Mre11-dependent double-strand break formation in *Saccharomyces cerevisiae* (reviewed in [5]), and uncoupled recombination and SC formation in *Caenorhabditis elegans* and *Drosophila melanogaster* (reviewed in [3], [4]). Meiosis in *C. cinerea* resembles that of most complex eukaryotes, with SC formation dependent on recombination, Mre11-independent double–strand break formation, and an average of one chiasma per chromosome arm [6]–[12].

The assembled 36.29 Mbp genome sequence of *C. cinerea* [6], [13], [14], has ∼ 13,400 open reading frames computationally predicted based on available EST data, comparisons with other fungal gene sets, and *ab initio* methods [6]. The availability of these data, combined with the tractability of *C. cinerea*, presents an ideal opportunity for the development and use of genomic technologies to investigate meiosis in this organism and compare meiotic transcription among eukaryotes.

The use of transcription profiles to infer gene function is particularly well-suited for the study of meiosis. While changes in transcription do not always correspond to functional effects, it was noted that timing of gene expression and protein function are often coincident during meiosis in *S. cerevisiae*, particularly for specialized processes such as recombination (reviewed in [15], [16]). Expression of meiotic genes likely requires tight control to prevent deleterious effects in other tissues; indeed, aberrant expression of meiotic genes has been implicated in mammalian cancer [16].

Insights into meiosis and spore development were provided by meiotic time courses in *S. cerevisiae* [17], [18] and *S. pombe* [19]. Meiosis and gametogenesis have also been profiled, by microarray and other methods, in several plant species (wheat, petunia, maize, rice) [20]–[23], silkworm [24], *D. melanogaster* [25], *C. elegans* [26], [27], and mammalian testis [16], [28]–[31]. These studies vary in their ability to distinguish meiotic stages due to the difficulties of dissecting purely meiotic tissues from larger structures such as anthers, and are hampered by the lack of synchrony, limiting the ability to sample defined meiotic stages. However, in common with the *S. cerevisiae* and *S. pombe* studies [17]–[19], transcriptional waves were apparent in a number of organisms [20], [28], [30], [31], with differential expression of genes essential for recombination, chromosome cohesion, and segregation noted in each species.

Comparative analysis of meiotic expression data from *S. cerevisiae* and *S. pombe* showed a lack of conservation of meiotic regulatory machinery, but nevertheless allowed definition of a “core meiotic transcriptome” of 75 genes [15]. This “core” group contained a number of previously characterized meiotic genes, such as *dmc1*, *rec8*, and *hop2*. However, several key meiotic genes are surprisingly absent from the list of core genes, including *spo11*, which encodes a key meiotic protein that makes double-strand breaks, suggesting that this list is not comprehensive.

*S. cerevisiae*, *S. pombe* and *C. cinerea* are highly divergent. Ascomycetes and basidiomycetes diverged ∼500–900 million years ago [32], [33], with the divergence of *S. cerevisiae* and *S. pombe* occurring shortly afterwards. Stimulation of entry into meiosis in both *S. cerevisiae* and *S. pombe* is induced by nutritional restriction, in contrast to the largely light-mediated induction of fruit body development and meiosis in *C. cinerea* [34]. The inclusion of *C. cinerea*, a more evolutionarily distant fungus with complex multicellular structure and differing meiotic cues, allows us to further investigate the evolutionary conservation of meiosis.

In this study, 70bp oligonucleotides were designed against the whole predicted *C. cinerea* transcriptome and used to assess transcript-level changes across a broad-scale time course encompassing meiosis in *C. cinerea*. The resultant transcriptional data were compared with similar data sets in *S. cerevisiae* and *S. pombe* to ask whether genes induced upon entry into meiosis and the transcript abundance during meiosis are conserved among the three fungal species.

## Results/Discussion

### A large proportion of *C. cinerea* genes change in expression during meiosis

**Figure 1:**
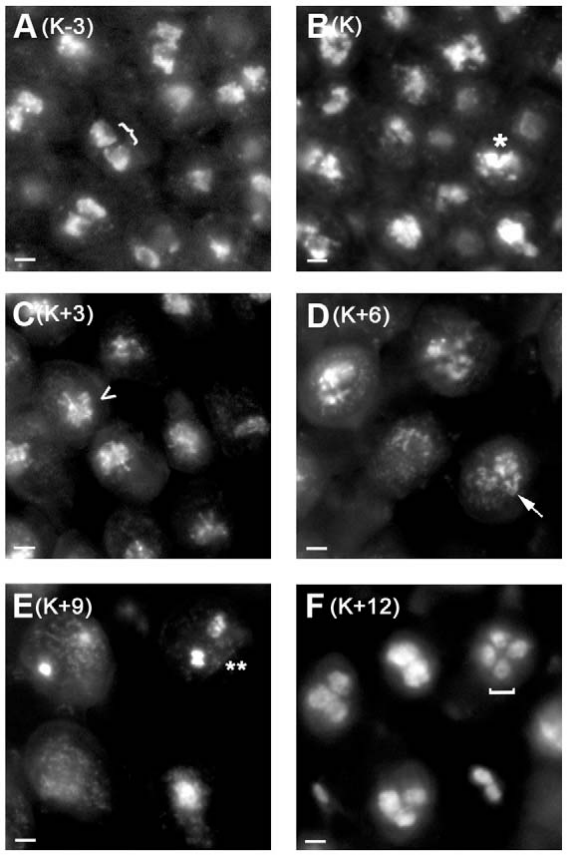
*C. cinerea* nuclei during meiosis. Adjacent basidia (meiotic cells) in gill tissue collected at 3-hour intervals from K−3 to K + 12 (A–F) and stained with DAPI. Two separate nuclei (}) are present prior to karyogamy (A), nuclei are fused or fusing (*) at karyogamy (B), chromosomes are condensing (<) at K+3 (C), are fully synapsed (arrow) at K+6 (D), and are undergoing the first meiotic division (**) at K+9 (E), and four nuclei are apparent (bracket) at K+12 (F). Fainter nuclei lie in a different focal plane. Note that the basidia become more widely spaced as meiosis progresses due to expansion of the underlying gill tissue. Scale  =  2 µm.

Oligonucleotide microarrays representing all the predicted *C. cinerea* genes were constructed and validated (see Materials and Methods), and used to investigate transcript level changes during meiosis. Gill tissue samples were taken from six time points spanning a 15-hour period. In *C. cinerea* dikaryons, haploid nuclei remain separate until just prior to meiosis, when they fuse (karyogamy). Gill tissue was collected at three hours before karyogamy (K−3; prior to meiotic DNA replication), at karyogamy (K), three hours after karyogamy (K+3; leptotene/zygotene), six hours after karyogamy (K+6; pachytene), nine hours after karyogamy (K+9; metaphase I), and twelve hours after karyogamy (K+12; tetrads have formed; Figure 1). cDNA populations derived from these samples were comparatively hybridized to microarrays, with a mixed sample as reference. Data were collected from four biological replicates for each time point. After data filtering and statistical analysis (see Materials and Methods), 2,851 probes (representing 2,721 genes) were found to exhibit changing expression during the meiotic time course. This is ∼20% of the 13,230 probes on the array, and corresponds with similar proportions of genes displaying differential expression in *S. cerevisiae* and *S. pombe* over similar time courses [17]–[19].

In several organisms, many meiotic genes are primarily expressed only in meiotic tissue (e.g. [26], [29], [35]–[38] and references therein). We identified genes expressed only in meiotic gill tissue in comparison with dikaryotic vegetative mycelia. In this comparison, 886 genes were expressed in meiotic gill tissue only, including genes with well-characterized roles in meiosis such as *hop1*, *spo11*, *rec8*, *mer3*, and *dmc1* (Table S1). Gene ontology (GO) enrichment analysis (using EASE within MeV [39], [40]; Materials and Methods) was used to identify over-represented gene functions within this group, revealing, as expected, enrichment of genes with meiotic function (Table S2).

### Clustering and gene ontology enrichment analysis of genes significantly changing during *C. cinerea* meiosis reveal transcriptional waves and distinct temporally regulated processes

**Figure 2:**
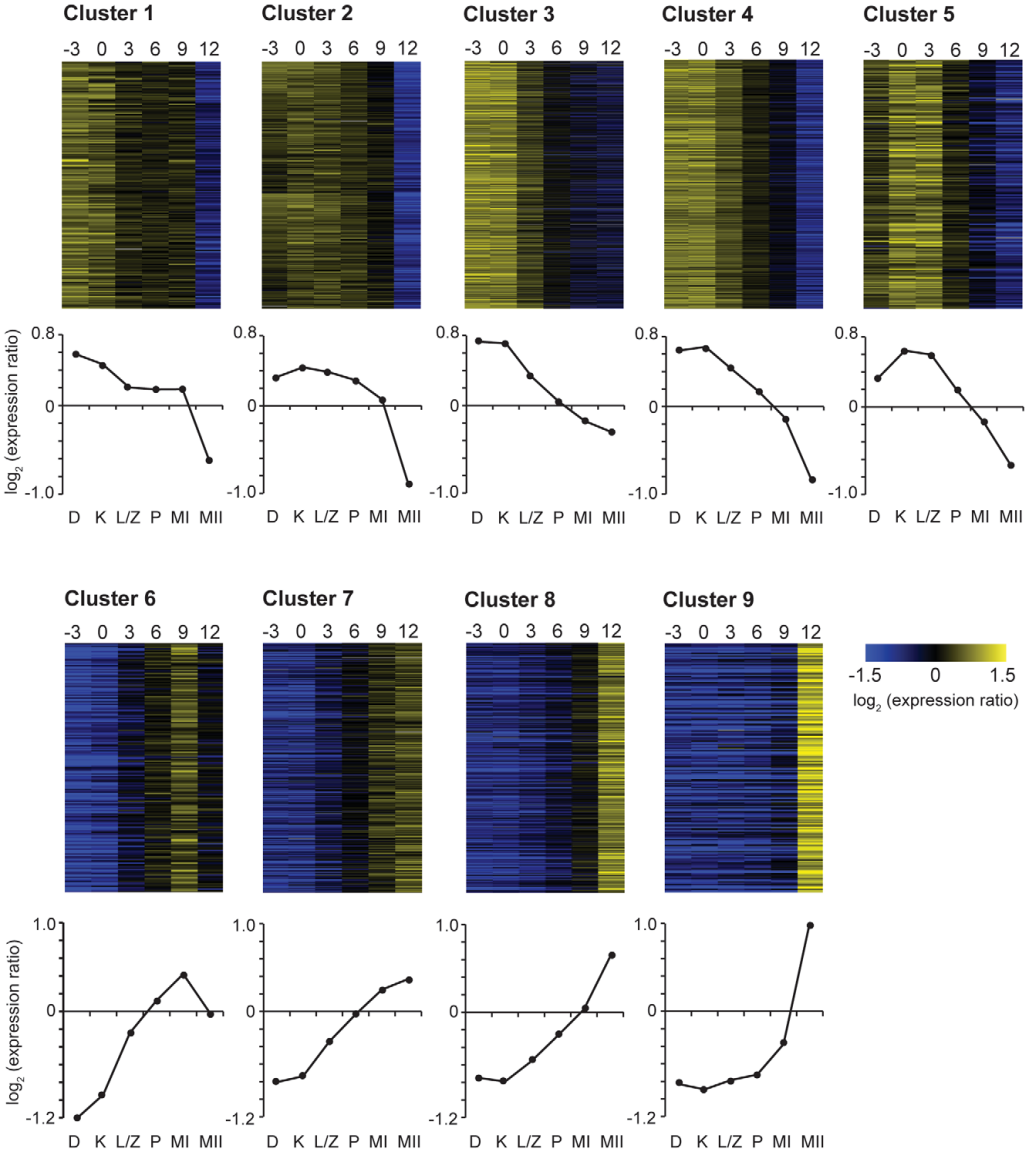
*C. cinerea* meiotic gene clusters. *C. cinerea* genes that changed in expression during the time course were grouped into nine clusters, as illustrated by heatmaps and average expression profiles of each cluster. Expression ratios are log_2_ transformed. Y-axis ratio scale markers  =  0.2. Time (hours) relative to karyogamy are shown at the top of the heatmaps. Meiotic stages are indicated at the bottom of the graphs; D  =  dikaryon, K  =  karyogamy, L/Z  =  leptotene/zygotene, P  =  pachytene, MI  =  first meiotic division, MII  =  second meiotic division.

Gene transcripts significantly changing during the *C. cinerea* meiotic time course were classified by clustering. Genes were K-means clustered using the Pearson correlation, which groups genes according to similarity in their temporal expression patterns, using a successive bifurcation strategy that removed user-choice from the resultant number of clusters (Materials and Methods, Figure S1). This strategy produced nine distinct gene transcript profiles (Figure 2), exhibiting successive “waves” of transcription, as reported for other organisms [16]–[31].

Gene ontology (GO) enrichment analysis of the nine clusters was used to identify over-represented gene functions within each of the nine *C. cinerea* clusters (Table S3). The nine clusters exhibit a clear difference in transcript profile between clusters 1–5 and 6–9, as indicated by the initial bifurcation (Figure 2). This is supported by the distinct classes of genes enriched in these clusters, which represents a broad switch from expression of genes required for the meiotic prophase I activities in early clusters, to expression of gill maturation and sporulation-related genes in later clusters (Table S3). This corresponds with a similarly dramatic transcriptional switch from “early” to “middle” gene expression, as observed in *S. cerevisiae* [17], [18].

Pre-meiotic DNA replication in *C. cinerea* occurs just prior to nuclear fusion [41]. Many aspects of DNA replication are well-conserved [42], such as the origin-recognition complex (ORC) and MCM2–7 complex, and these genes are expressed primarily in early clusters 1–3 (Table S1). Clusters 1–3 are enriched in functional categories of genes involved in early meiotic processes (Table S3); DNA replication is reflected in categories such as nucleic acid binding. Cluster 2, which exhibits a more prolonged transcript presence than other clusters, is enriched for regulation and organization of the cytoskeleton. This is likely to be important for karyogamy, organization of the meiotic spindle, and segregation of chromosomes; these processes span the entire time course, explaining the prolonged requirement of these transcripts. RNA splicing functions are also enriched in cluster 2, which is notable because control of splicing has been implicated in meiotic regulation [43], [44]. All the genes encoding components of the cohesin complex (*scc3*, *smc1*, *smc3* and the meiosis-associated factor *rec8*) are present in cluster 3. Cohesin holds sister chromatids together during meiosis, and primarily loads early, during replication. The gene encoding Spo11, which initiates recombination through its formation of double-strand breaks [45] is also in cluster 3. This suggests that cluster 3 may be a source of promising candidates for early-acting meiotic genes.

We noted a massive enrichment of genes involved in ribosome production, translation, protein catabolism, and ribosomal RNA processing in cluster 4. In *S. cerevisiae*, ribosomal protein genes are repressed on entering meiosis, with a subsequent increase in expression during sporulation, reflecting the starvation conditions required to induce meiosis in this organism [17]. In *C. cinerea*, transcript levels of ribosomal protein genes are relatively high until karyogamy, after which a gradual decline is observed, with no subsequent increase of transcription. Ribosomal degradation prior to meiosis and subsequent resynthesis during meiosis or sporulation have been previously noted in *S. cerevisiae* and *Chlamydomonas reinhardtii* (reviewed in [46]), and ribosomal turnover is implicated in regulation of cell growth and proliferation in *Xenopus laevis* and *D. melanogaster* (reviewed in [47]). Enhanced expression of ribosomal genes in *C. cinerea* at K-3 and K may be in preparation for meiosis and for the massive, rapid cellular expansion in the gills and fruit body over the timescale examined in this time course.

Cluster 5 genes exhibit intermediate transcript levels prior to karyogamy, with increased levels of expression during nuclear fusion and leptotene/zygotene, after which transcripts decrease rapidly. Cluster 5 is highly enriched for genes known to be involved in meiotic processes such as damaged-DNA binding, mismatch repair, and DNA modification (Table S3). Characterized genes in this cluster include those critical for key meiotic events such as strand exchange (*dmc1*, *rad5*, *rdh54*), axial element formation and synapsis (*hop1*), and crossover formation (*msh5*, *mlh1*). Several of the genes expressed in cluster 5 play key roles in meiosis in other organisms (as summarized in [37]), making this cluster a rich source for exploration of meiotic gene candidates.

Clusters 6–9 are enriched in genes required for spore formation. We observed a progressive shift from expression of biosynthetic genes, which may play a role in gill expansion due to carbohydrate acquisition and vacuolation (e.g. fatty acid synthesis in cluster 6, sugar and energy reserve synthesis in cluster 7) to those involved in formation of spore structure and spore packaging (e.g. cell wall biogenesis in cluster 8 and extracellular polysaccharide and carbohydrate transport in cluster 9) as well as preparation for spore germination (spore germination associated genes in cluster 8, and those involved in perception of external stimuli in cluster 9). A comparative analysis of spore formation, although potentially of great interest, is beyond the scope of this study.

### Meiotic function genes are more conserved in their induction and expression patterns than genes not known to be meiotic

Previously, meiotic genes in *S. cerevisiae* and *S. pombe* were found to be more likely to be co-induced than a control set of genes with orthologs in *S. pombe* that were induced in one *S. cerevisiae* strain but not another [19]. We wished to ask if meiotic genes are also more likely to be co-induced than non-meiotic genes in comparisons among the two yeasts and *C. cinerea*.

To determine which genes are induced upon entry into meiosis in *C. cinerea*, we compared gene expression during vegetative dikaryotic growth to expression in meiotic gill tissue at K-3. We observed 886 genes to be expressed only in gill tissue, with a further 3,621 genes expressed in vegetative tissue but significantly induced upon entry into meiosis. To ask whether genes meiotically induced in *C. cinerea* are also induced upon meiotic entry in *S. cerevisiae* and *S. pombe*, we identified single copy, unambiguous, putative orthologs (henceforth referred to as “orthologs”; see Materials and Methods) and compared their patterns of induction. Transcript level changes upon meiotic entry in *S. cerevisiae* and *S. pombe* were determined from previously published microarray data [17], [19], and induction of the meiosis-associated gene *spo11* was used as a control indicator of the transition between non-meiotic and meiotic cells. Transcript level changes in *S. cerevisiae* and *S. pombe* were compared with one another and with the significant changes between vegetative tissue and K-3 in *C. cinerea*.

Orthologs and expression data were available in all three fungal species for 2,006 genes. Genes were assigned to “meiotic function” (MF) or “no known meiotic function” (NKMF) categories as defined by the *Saccharomyces* Genome Database [48] and the Gene Ontology [49] (Table S1). Of the 2,006 pertinent genes, significant induction on entry to meiosis was observed for 1,046 *C. cinerea* genes. In the yeasts, 829 *S. cerevisiae* genes and 869 *S. pombe* genes were induced. We considered the 829 genes induced in *S. cerevisiae*, as this is the maximum number of genes with the potential to be induced in all three species, and asked how many were induced upon entry into meiosis in all three species for the MF and NKMF classes. We observed that 50 of the 119 MF genes were induced in all three fungal species, while only 169 of the 710 NKMF genes were co-induced (p<1×10^−^4, Fisher's Exact test). The names and putative functions of genes in the MF_co-induced, MF_not_co-induced, and NKMF_co-induced categories are listed in separate tabs in Table S1.

The 50 commonly induced MF gene set contains a number of genes known to be crucial for meiosis, such as all three genes of the Mre11 complex (*mre11*, *rad50* and *xrs2/nbs1*), genes encoding strand invasion proteins (*dmc1* and *rad51*), and genes encoding meiosis-associated proteins (*spo11*, *rec8*, *hop1 and dmc1*). This suggests that coordinate induction of genes across multiple species may prove to be an indicator of meiotic function; the inclusion of *C. cinerea* as a comparator clarifies those genes that are likely evolutionarily conserved in their meiotic behaviour. Several of the genes that are coordinately induced in all three species but currently have no known meiotic function are involved in spindle formation, chromosome segregation or DNA-metabolic processes, and may yet prove to be important in meiosis (Table S1).

Comparison of genes coordinately induced on entry into meiosis is necessarily a binary approach, asking “on/off” questions that do not query the changes in transcript level through a time course. A complementary approach is to compare the temporal transcript profiles of genes during meiosis. Comparative studies of mammalian gametogenesis asked whether genes were conserved in their relative expression patterns in distinct pre-meiotic, meiotic, and post-meiotic tissues in rat and mouse (∼23 million years divergent [50]), and found that correlated genes were enriched for reproductive function [15], [28], [29]. The availability of time course data describing meiosis in three different fungal species affords us the opportunity to ask if conservation of transcript profile can also be observed within meiotic cells in these more diverged organisms. This may also highlight similarities and differences in meiotic process not observed by examining coordinate induction.

**Figure 3:**
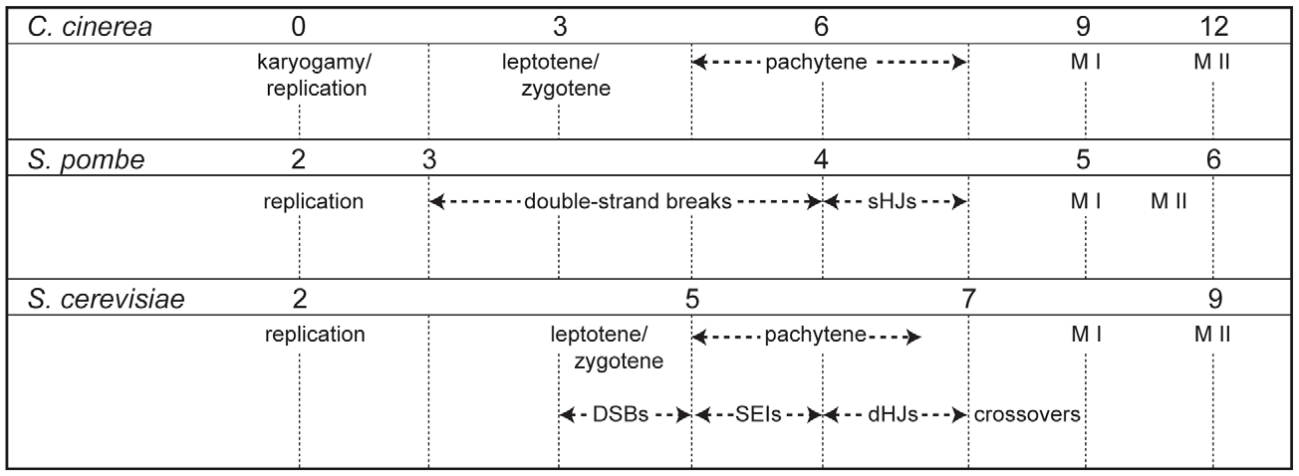
Timing of meiotic events in *C. cinerea*, *S. pombe*, and *S. cerevisiae*. Time points used to examine meiotic transcription in *S. pombe* [19] and *S. cerevisiae* [17] were aligned with those used in *C. cinerea* according to observations from existing time courses [2], [17]–[19], [85]–[87]. Time points are shown as hours after switching to sporulation media (*S. pombe* and *S. cerevisiae*) or hours after karyogamy (nuclear fusion) in *C. cinerea*. Aligned time points are indicated with dashed lines. sHJs  =  single Holliday junctions, DSBs  =  double strand breaks, SEIs  =  single end intermediates, dHJs  =  double Holliday junctions, M I  =  first meiotic division, M II  =  second meiotic division.

We examined the 2,721 genes with significantly changing transcript levels in *C. cinerea*, and found *S. cerevisiae* and *S. pombe* orthologs and corresponding expression data for 743 genes [17], [19]. Meiotic progression in the three fungi differs with respect to the overall time required for completion of meiosis, and the duration of certain stages within the meiotic program. Thus, in order to compare meiotic transcript profiles in the three species, we aligned expression data according to previously described meiotic landmarks and defined an eight-point time course (Figure 3). Data were unavailable for all three species at every stage defined; in these cases, expression data were interpolated by averaging the expression from flanking time points.

**Figure 4:**
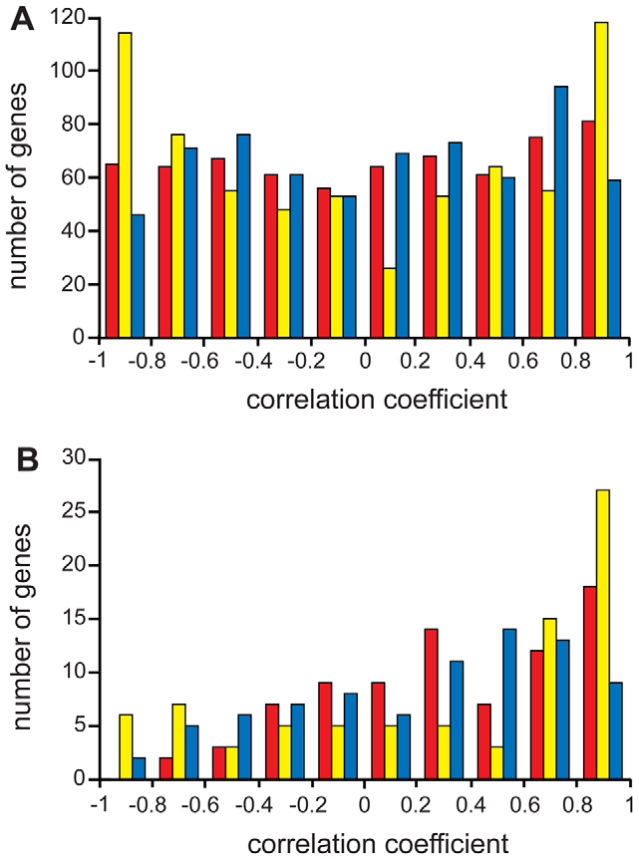
Distribution of gene expression profile correlation coefficients. Distributions of pair-wise correlation coefficients for *C. cinerea vs. S. pombe* (red), *C. cinerea vs. S. cerevisiae* (yellow) and *S. pombe vs. S. cerevisiae* (blue) for genes with no known meiotic function (A) and for meiotic function genes (B). Note that the meiotic function genes (B) have relatively more pairwise expression profiles with positive correlation coefficients than genes with no known meiotic function (A).

The 743 orthologs were again divided into MF genes (81) and NKMF genes (662). For each orthologous gene, the transcript profiles were compared for each of the three possible interspecies pair-wise combinations (i.e., *Ccin vs. Scer*, *Ccin vs. Spom*, *Scer vs. Spom*), and correlation coefficients (*r*) were generated. In all comparisons, more transcript profiles are well-correlated (*r*>0.5) for MF genes than NKMF genes (*Ccin/Spom*, 44% vs. 28%; *Ccin*/*Scer*, 54% vs. 30%; *Spom*/*Scer*, 32% vs. 29%), and the correlation value distributions (Figure 4) were significantly different when MF genes were compared with NKMF genes (Mann-Whitney-Wilcoxon test: *Ccin*/*Spom*, W = 38049, *p*<0.0001; *Ccin*/*Scer*, W = 37655, *p*<0.0001; *Scer*/*Spom*, W = 34760, *p*<0.0112). Thus, the transcript profiles of MF genes are more highly conserved than those of NKMF genes. Fifty-two genes of the NKMF class are well-correlated in all three pair-wise comparisons (and six of these genes are also coinduced in all three species); these subsets (Table S1) provide an interesting pool of candidates that may have additional, as yet uncharacterized, meiotic functions.

**Figure 5:**
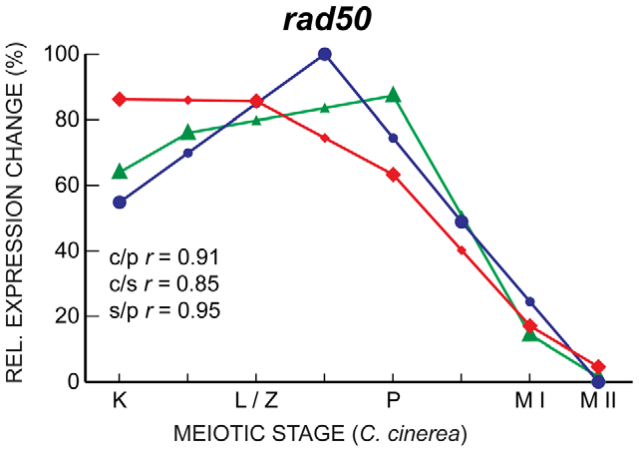
*rad50* expression is well-correlated in *C. cinerea*, *S. pombe*, and *S. cerevisiae*. Expression profiles of *rad50* in *C. cinerea* (red ⧫), *S. cerevisiae* (blue •) and *S. pombe* (green ▴) are shown across eight time points for which biological stages are comparable. Symbols representing interpolated data points are smaller. Expression profile correlation coefficients (*r*) are shown for *C. cinerea vs. S. pombe* (c/p), *C. cinerea vs. S. cerevisiae* (c/s) and *S. cerevisiae vs. S. pombe* (s/p). *C. cinerea* meiotic stages are as follows: K, karyogamy; L/Z, leptotene/zygotene; P, pachytene; M I, just after first meiotic division; M II, just after second meiotic division. Corresponding meiotic stages for *S. pombe* and *S. cerevisiae* are shown in Figure 4. To allow visual comparison of profiles from different species, expression data are adjusted to show relative expression change across the time course for each species.

Given that MF genes are enriched both for coordinate induction on entry to meiosis and transcript profile correlation though meiosis, we noted some surprising differences in induction and correlation in some meiotic genes, highlighting the complementary value of both these types of analysis. Of genes coordinately induced in the three species, several do not exhibit well-correlated transcript profiles. Of the 50 coordinately induced MF genes, only 41 are well correlated between at least 2 of the three species, with only a single gene (*rad50*) coordinately induced and well-correlated in all three species (Figure 5), indicating that transcript profile conservation reveals additional information about meiotic regulation; coordinate induction does not predict transcript profile correlation or vice versa. Meiotic genes may be expected to be all induced upon meiotic entry, but their subsequent expression behavior may be able to inform us about the different ways meiosis is achieved in different organisms.

**Figure 6:**
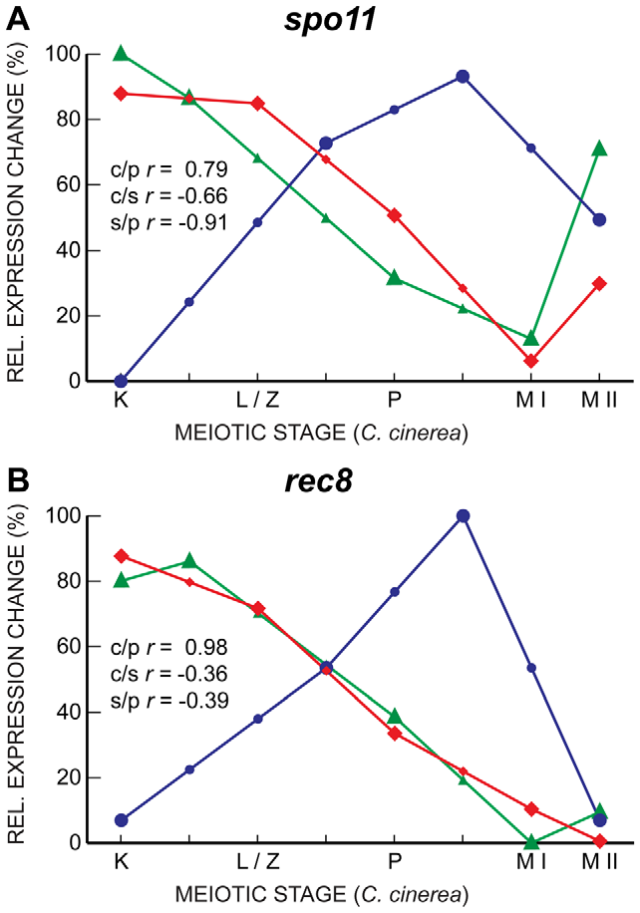
Gene expression in *spo11* and *rec8* are well-correlated only between *C. cinerea* and *S. pombe*. Gene expression profiles of *spo11* (A) and *rec8* (B) are shown for *C. cinerea* (red ⧫), *S. cerevisiae* (blue •) and *S. pombe* (green ▴) as in Figure 5.

Interestingly, despite their well-characterized meiotic roles, and although they are induced upon entry into meiosis in all three fungal species, the transcript profiles of *spo11* and *rec8*, which encodes a meiosis-associated cohesin subunit [51], [52], are well-correlated only between *C. cinerea* and *S. pombe*, with *S. cerevisiae* expression peaking late in meiosis, just before the first meiotic division, later than the timing of the corresponding protein activity (Figure 6). Other genes essential for meiosis, such as *hop1* and *dmc1*, also exhibit a similar late transcript peak in *S. cerevisiae*. This unexpected lack of correlation may indicate additional or differing functions for some meiotic proteins; for example, Spo11 forms meiotic double-strand breaks independently of the Mre11 complex in *C. cinerea* and *S. pombe*, but in an Mre11-dependent manner in *S. cerevisiae* [5], [53], [54]. Alternatively or additionally, post-transcriptional regulation might be more prevalent in core meiotic genes in *S. cerevisiae*. For example, alternative splicing of introns is disproportionately involved in meiotic gene regulation when compared to other biological processes in *S. cerevisiae* [43].

**Figure 7:**
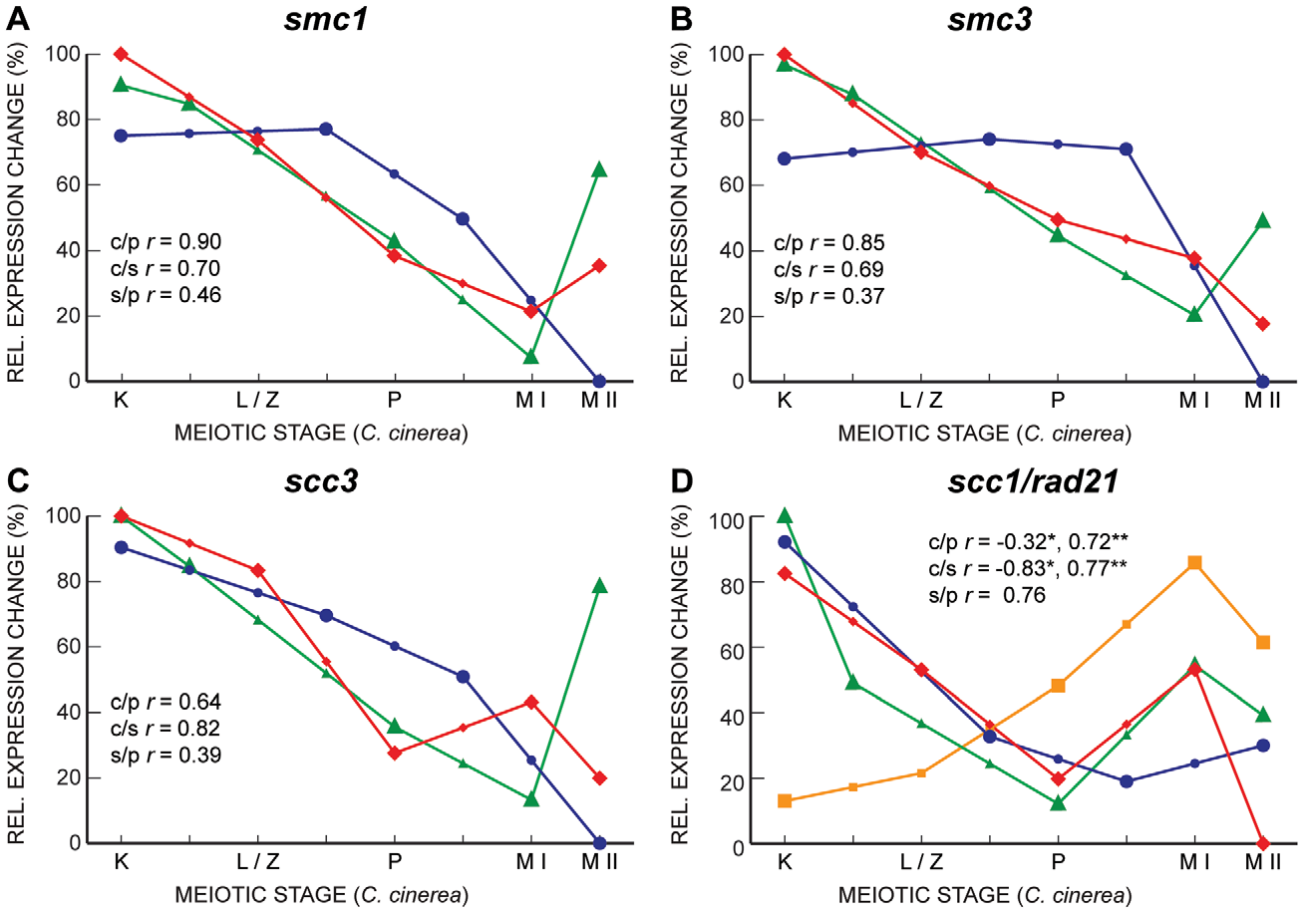
Gene expression profiles of cohesin subunits are well correlated between *C. cinerea* and *S. cerevisiae*, and between *C. cinerea* and *S. pombe*. Gene expression profiles of *smc1* (A), *smc3* (B) and *scc3* (C) are shown for *C. cinerea* (red ⧫), *S. cerevisiae* (blue •) and *S. pombe* (green ▴) as in Figure 5. Panel D shows expression profiles of *scc1/rad21* in *S. cerevisiae* (blue •) and *S. pombe* (green ▴), as well as the two homologs in *C. cinerea*, *rad21.1* (orange ▪) and *rad21.2* (red ⧫). Correlation coefficients are as indicated for *rad21.1* (*), and *rad21.2* (**).

Other genes exhibit the correlated transcript profiles expected given the roles of their proteins. For example, the genes encoding three members of the cohesin complex, Smc1, Smc3, and Scc3, are all well-correlated between *C. cinerea* and *S. cerevisiae*, and somewhat correlated between *S. cerevisiae* and *S. pombe* (Figure 7). Single genes encode the cohesin Scc1 (*S. cerevisiae*)/Rad21 (*S. pombe*) in the yeasts, whereas *C. cinerea* has two homologous genes encoding this protein. For one of these, *rad21.2*, the transcript profile is well-correlated with those of its *S. cerevisiae* and *S. pombe* homologs. The Rad21.2 protein is found exclusively in meiotic tissue (Palmerini *et al*., in preparation). In contrast, *rad21.1* displays a very different transcript profile (Figure 7). The Rad21.1 protein is the only mitotic kleisin in *C. cinerea* (Palmerini *et al.*, in preparation) and its RNA abundance spike late in meiosis may reflect transcription in preparation for the first post-meiotic mitosis.

**Figure 8:**
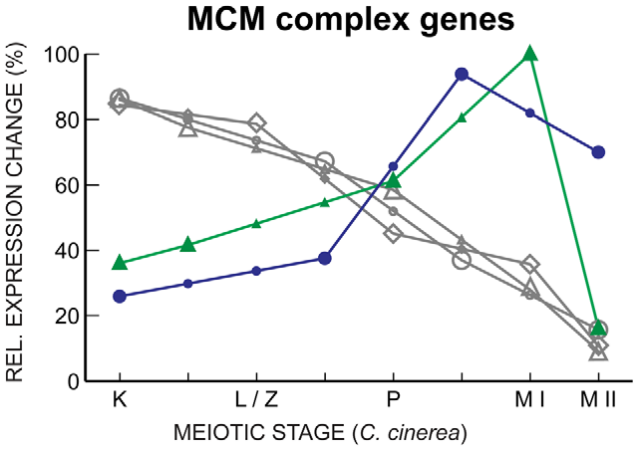
MCM complex gene expression declines through meiosis, with key exceptions. Gene expression profiles of *mcm7* in *C. cinerea* (gray ⋄), *S. cerevisiae* (gray ○) and *S. pombe* (gray ▵), and *S. pombe mcm6* (green ▴) and *S. cerevisiae mcm5* (blue •) are shown as in Figure 5.

We also noted an unusual expression pattern of some MCM complex genes. This complex, composed of MCM2–7, is involved in replication, and thus one would expect these genes to be expressed early and coordinately. Most of the genes are indeed expressed in such a manner, with the exception of *S. pombe mcm6* and *S. cerevisiae mcm5*, which have very similar, late-peaking, expression profiles (Figure 8). *C. cinerea mcm2* also exhibited this late expression profile, but the change through meiosis was not statistically significant. In *Drosophila melanogaster*, *mcm5* mutants are defective in the resolution of meiotic double-strand breaks to crossovers [55] raising the possibility of a similar meiotic role for *mcm5* in *S. cerevisiae*. The similar expression patterns of *S. pombe mcm6* and *C. cinerea mcm2* suggest this additional role may not be confined to a specific MCM subunit.

Previous studies have shown both conservation and apparent divergence in proteins required for meiosis [37], [38], [56]–[61]. Our work shows that, for proteins whose primary sequence is conserved enough for homology to be recognized, gene expression profiles throughout meiosis are significantly conserved. That this conservation occurs across >500 million years of evolution suggests that meiosis is more conserved than hitherto recognized. Based on our data and existing criteria, we propose an expanded inventory of genes involved in meiosis (Table S4).

We also predict that additional conservation of meiotic genes will be found as the algorithms for detecting homology become more sophisticated. This has practical implications, in that previously uncharacterized genes with meiotic roles could be identified both by similarity of expression profile to known meiotic genes within an organism (i.e., Figure 2) and by conservation of expression profile across organisms. For example, *ubc9*, which is induced upon entry into meiosis in the three fungi, has well correlated expression profiles in a comparison between *C. cinerea* and *S. pombe*, and a late peaking expression profile similar to that of *spo11* and *rec8* in *S. cerevisiae*. Ubc9 has no known meiotic role in the yeasts but is involved in sumoylation during meiosis in *C. cinerea* [62], [63]. Genes with orthologs in the *C. cinerea* meiotic cluster 5 with no currently identified meiotic function (53) are also compelling candidates for study. Of these genes, four, including those encoding two transcription factors (Hir1 and Tfb2) are coordinately induced in all three fungal species. An additional six genes are well-correlated in pair-wise comparisons in all three fungi. In addition, given the sequence divergence of many meiotic genes, such as those encoding synaptonemal complex components, a proportion of the genes in cluster 5 with no currently apparent orthologs are likely to have meiotic roles. This is illustrated by *bad42*, which has a critical meiotic role in *C. cinerea* meiosis but has no known orthologs [64].

It is logical that meiotic processes must be tightly controlled to avoid deleterious effects of renegade gene expression (e.g. [16]). The broad conservation of meiosis opens up interesting possibilities for the study of this process in different organisms. Thus, protein function can be inferred from studies in different species and the exploitation of the benefits of various study organisms, such as the elegant cytology and uncoupled recombination and SC formation in *C. elegans* and *D. melanogaster*; the tractability of *S. cerevisiae* and *S. pombe*; and the synchronous meiosis, facile screening, and prolonged prophase in *C. cinerea*. In addition, the striking differences in expression pattern between species for necessarily tightly controlled genes such as *spo11* indicate differences in meiotic gene regulation, highlighting the value of different types of analysis.

## Materials and Methods

### Design, production and validation of a *Coprinopsis cinerea* 70-mer oligonucleotide microarray

To produce a microarray for *C. cinerea*, 70-mer oligonucleotides were designed against the then-available ∼12,500 predicted gene sequences using ArrayOligoSelector [65] in successive design rounds, initially using a secondary target binding energy cut-off of −25 kcal/mol and relaxing where necessary. Where possible, oligonucleotides were 3′-biased and filtered to maximize the chance of specific hybridization; using FastA [66] and BLASTn [67], oligos were discarded that had a secondary match with >85% similarity, or a secondary hit with >20bp contiguous match [68]. Oligos that spanned more than 1 intron were also discarded. Oligonucleotides were also designed against repeat elements (without secondary match filtering), known *C. cinerea* genes, and a small subset of EST sequences that were not represented by gene predictions. In total, 13,230 probes were designed, with 1,061 of the predicted genes represented by more than one probe. A total of 12,104 probes representing 11,746 genes of the Jan06m300_GLEAN prediction set were designed, plus an additional 165 probes representing 162 aug_GLEAN predicted genes. Some predictions from the Jan06m300_GLEAN set do not have oligos, as a proportion were shorter than the 70bp oligo length, and many corresponded to repeated elements and were removed. Some ESTs were not represented by gene predictions, and 390 additional probes represent unique ESTs. A total of 467 probes were designed to distinct repeated elements. Also, 48 probes represent mating factor genes from different *C. cinerea* strains that are thus not present in the sequenced strain. The 56 remaining probes were designed against existing NCBI *C. cinerea* sequences, which often had small variations in comparison with the sequenced strain. These slightly mismatched oligos were therefore not mapped to gene predictions. Oligonucleotides were resuspended in 3×SSC at a final concentration of 20µM, and were printed onto amine-coated glass slides (Cel) by the Center for Genomics and Bioinformatics, Indiana University. Microarrays were UV-crosslinked at 450 mJ prior to hybridization. To assess probe performance, data from the genes represented on the array by more than one probe were compared and found to be well-correlated, as determined by a Pearson product-moment test (correlation coefficient  =  0.89); data from single probes are thus highly reliable (Figure S2).

Array results were validated using qPCR data for a number of genes, including genes of high and low expression, genes with steady expression throughout the time course, and genes of known function in *C. cinerea* (Figure S2, Table S5). Data were normalized against an average of all the data points for a given primer pair or probe in order to render array and qPCR data directly comparable. Array and qPCR data were determined to be well-correlated as determined by a Spearman rank order test, which gave a correlation coefficient of 0.84; this compares favorably with similar analyses of other microarray datasets [69]. Furthermore, array results for characterized *C. cinerea* genes correspond with published northern data [8], [11], [12], [70]–[73]. Statistical analysis for comparison of array and qPCR data, and comparison of multiple probe gene data, were done using Minitab (http://www.minitab.com).

### Fungal strains and culture conditions

*C. cinerea* wild-type monokaryon strains J6;5-5 and J6;5-4 [74] were incubated at 37°C on YMG media. These strains were mated, and the resulting dikaryon subcultured into fruiting tubes, grown at 37°C for 2 days, and then moved to 25°C under a regime of 8 hours dark, 16 hours light, as previously described [75]. Fruiting initials emerged after *ca.* 7 days and synchronous mushroom caps were harvested at three hours prior to karyogamy (K−3), karyogamy (K), and 3, 6, 9, and 12 hours post-karyogamy (K+3, K+6, K+9, K+12) and frozen with liquid nitrogen. For vegetative tissue, fragmented dikaryotic or monokaryotic mycelia were used to inoculate static 20ml liquid YMG cultures. Static cultures were incubated at 37°C for 2 days, then used to inoculate 100ml YMG. These larger cultures were shaken at ∼150 rpm at 37°C for 2 days, then harvested with a Buchner funnel and immediately frozen with liquid nitrogen.

### RNA isolation and array hybridization

Mushroom gill tissue was excised from 7–10 fruiting bodies per time point and, after removal of veil tissue, immediately frozen in liquid nitrogen. Dikaryotic and monokaryotic vegetative mycelia were harvested through a Buchner funnel. RNA was extracted from all the tissue collected using the RNeasy Plant Mini Kit (Qiagen) according to the manufacturer's instructions. The RNA yield typically ranged from *ca.* 100µg-1mg, of which 20µg was used per array hybridization. First strand cDNA was synthesized and labeled with Alexa-fluor dyes using the Superscript Indirect cDNA Labeling System (Invitrogen) as described. For gill samples, two-channel hybridizations were performed, comparing a time point (test) sample with a reference mixture of time point samples, to maximize array resolution. Due to sample limitations, the reference consisted of cDNA from the latter 4 time points only. Four biological replicates of each time point were tested, incorporating dyeswaps. Vegetative dikaryotic and monokaryotic mycelia were directly compared in two-channel hybridizations. Microarray slides were blocked prior to hybridization with 5×SSC, 0.1%SDS, 0.1mg.ml^−1^ BSA at 42°C for 45 min, after which slides were rinsed twice in room temperature 0.1×SSC for 5 min each, followed by a final 10s rinse in water. Slides were dried by centrifugation for 1min in a Labnet C1303 slide spinner. Arrays were placed in Corning hybridization chambers and mSeries Lifterslips (Erie Scientific) placed over the array grids. Labeled cDNAs were combined in 50µl with 30% deionized formamide (Ambion), 5×SSPE, 0.2% SDS, 1µg.µl^−1^ tRNA (Invitrogen), 40ng.µl^−1^ oligo dA (Invitrogen). The hybridization mixture was heated at 100°C for 2min, cooled to 25°C, and applied to the microarray slide. Slides were hybridized for 16 hours at 42°C, then washed for 5 min each in 1×SSC, 0.03%SDS (37°C), then 0.2×SSC (ambient temperature), and finally 0.1×SSC (ambient temperature). Slides were dried by centrifugation, as previously, and scanned immediately.

### Quantitative reverse-transcription polymerase chain reaction

Primers for quantitative RT-PCR were designed using Primer Quest (idtdna.com) with an amplicon size of 200–250bp, target Tm of 59°C with no more than 2°C Tm difference between primer pairs, 50% GC, target primer length 24nt. Eleven genes were examined with qPCR (Table S5). RNA samples were treated with Turbo DNase (Ambion) and quantified using a Nanodrop Spectrophotometer. Equal quantities of RNA were reverse transcribed using qScript cDNA SuperMix (Quanta). The resulting cDNA was diluted 16-fold for use in qPCR. PCR was performed in triplicate, in 15µl reactions using PerfeCTa SYBR Green FastMix Low ROX mix (Quanta), 150nM primers (final concentration) and 5µl cDNA. Duplicate qPCR reactions of a standard curve comprising a 4-fold dilution series of genomic DNA or mixed cDNA was performed alongside test reactions, as well as no template and no reverse transcriptase controls. Reactions were assembled in 96 well plates (Stratagene) and performed on a Stratagene MX3000P instrument, with the following cycle: 95°C, 10 min; (95°C, 30 s; 59°C, 1 min; 72°C, 1 min)×40 cycles (qPCR); 95°C, 1 min; 55°C, 30 s; 95°C, 30s (melt curve analysis). Transcript copy number was estimated from the standard curve.

### Microarray data capture and analysis

Microarray slides were scanned using a GenePix 4200A scanner (Molecular Devices). Spots were identified using GenePix Pro software (Molecular Devices) and manual inspection. Scans were quality assessed using the Basic Hybridization Analysis R script (http://cgb.indiana.edu/downloads/1). Spots were manually flagged to be excluded from analysis if there were areas of poor quality such as scratches or dust. Spots were also flagged for omission, using GenePix software, if they fulfilled any of the following criteria: manually flagged, buffer only spots, spots not found, percentage of saturated pixels in both channels >3, percentage of pixels above background plus 1 standard deviation in both channels<60, spot pixels<40.

Data normalization and filtering were performed using the Bioconductor [76] (http://www.bioconductor.org/) packages marray and OLIN [77] as well as custom scripts. OLIN [77] was used for intra-slide normalization and log_2_ transformation.

For statistical analysis of meiotic time course data, data from a given probe were included only if they fulfilled one or both of the following criteria. First, data for a given oligonucleotide were included if two out of four biological replicates for every time point contained data for both probes. This criterion was chosen to avoid the inclusion of data from single replicates (i.e., for which none or only one replica produced data of acceptable quality). However, data for probes that had robust data for all four replicates for at least one time point were also included, whether or not they fulfilled the first criterion. Of the 13,230 array probes, 8,413 fulfilled one or both of these criteria, of which 286 fulfilled only the first criterion, 941 fulfilled only the second, and 7,186 fulfilled both. Significance Analysis of Microarrays (SAM) software [78] was utilized to determine which genes were changing in expression during the meiotic time course progression, using a false discovery rate (FDR) of 10%. This cut-off encompassed most of the *C. cinerea* genes previously characterized as having differential expression during meiosis. Using the 10% FDR cut-off, 2,851 probes (representing 2,721 genes) exhibited differential expression over the 15-hour time course.

Gene expression in vegetative dikaryotic mycelia was compared to K-3 expression by combining single channel dikaryotic data and single channel K-3 expression data. Single channel data from three independent dikaryotic replicates were scaled to the same median, and the data averaged. Data for a given spot were excluded if only one replicate was present. The average dikaryotic data were combined with individual K-3 replicates within four separate GPR files. Data were flagged, OLIN normalized, and analyzed with SAM as described above.

### Data clustering and gene ontology

All clustering and Gene Ontology (GO) analysis was performed within the MeV framework [39]. Genes assessed to be differentially expressed across the time course by SAM were clustered using the K-Means Support function, with the Pearson correlation as similarity measure [79] and a successive splitting strategy similar in approach to [80]. Within K-Means Support, the clustering algorithm was performed 100 times, and genes were assigned to a cluster if they fell within the same group for at least 90 of these iterations. Initially, the full set of 2,851 genes was divided into two clusters, after which each of these resultant clusters was further bifurcated repeatedly. Terminal clusters were those that could not be further divided at the 90% level. Nine robust clusters resulted from this strategy.

After removal of duplicate probes to the same gene in a cluster, EASE analysis [40] was performed to determine enrichment of gene classes by gene ontology. Gene ontology terms assigned to genes from the Jan06m300_GLEAN prediction set (11746 of which are represented on the array) were used for EASE analysis. Gene ontology classes were deemed enriched if there was >1 gene of that class in the cluster and the Fisher's Exact statistic was <0.01.

### Identification of putative orthologs of *C. cinerea*, *S. cerevisiae, and S. pombe*

OrthoMCL [81] was run using WU-BLASTP with significance cutoff of 1e^−4^, with Smith-Waterman post-alignment, and low-complexity filtering with seg+xnu on the protein sets of *Coprinopsis cinerea* (v2, Jan 2009; Broad Institute and [6]), *Schizosaccharomyces pombe* (downloaded Feb 19, 2009; GeneDB) and *Saccharomyces cerevisiae* (downloaded Feb 20, 2009; SGD). Briefly, OrthoMCL uses the Markov Clustering approach [82], [83] to identify clusters of genes by similarity with additional correction for paralogous gene distances. An inflation value of 1.5 was used to build the clusters. It has been shown to be one of the more accurate approaches to identifying orthology [84]. The OrthoMCL clusters containing *C. cinerea* genes and single-copy members of described meiotic genes from *S. pombe* and *S. cerevisiae* were used to identify the complement of these genes in *C. cinerea*.

### Comparative analysis of gene expression in different fungi

For comparison of genes induced on entry to meiosis in *C. cinerea*, *S. cerevisiae* and *S. pombe*, we identified the time-interval during which *spo11* was initially expressed. For *C. cinerea*, this was the interval between vegetative dikaryotic growth and K−3. Significantly changing genes were identified using SAM as for the meiotic time course. For *S. cerevisiae*, the suitable interval was between time 0 (the time of nutritional restriction to stimulate meiosis) and 1 hour subsequently [17]. In *S. pombe*, *pat1* mutant vegetative cells were preconditioned for meiosis by starving overnight, before induction of meiosis by a temperature shift at time 0 [19]. Initial expression of *spo11* (and other meiotic genes) occurred in the starvation interval, between vegetative and time 0.

To compare expression profiles of genes throughout meiosis, expression data from time courses of *C. cinerea*, *S. cerevisiae* and *S. pombe* were compared using the Pearson correlation within Excel. Genes were compared only if single putative orthologs were found in all three fungal species. Gaps in biological expression data were interpolated by taking the mean of flanking data points. The Mann-Whitney-Wilcoxon test was used to determine whether the distribution of correlation coefficients differed between groups of genes. Statistical comparisons were performed using Minitab (http://www.minitab.com). “Meiotic function” genes were defined by using “meiosis”, “DNA-replication”, “DNA repair”, and “recombination” to search the *Saccharomyces* Genome Database [48] and the Gene Ontology [49].

### Microscopy

Images of meiotic gill cells were obtained as previously described [54].

### Data

Raw microarray expression data obtained in this study are accessible in the Gene Expression Omnibus database (GEO), with accession numbers GSE13731 and GSE18540. Table S1 provides summary data on all array probes.

## Supporting Information

Figure S1 Schematic of gene clustering strategy. The 2,851 probes identified as changing in expression by SAM at an FDR less than 10% were grouped into clusters using a successive bifurcation strategy, as illustrated.
(0.02 MB PDF)

Figure S2 Assessment of microarray probe performance. Array probes were tested for reliability by comparison of expression with a second probe for the same transcript (A; correlation coefficient  =  0.89) and by comparison with qPCR expression data (B; correlation coefficient  =  0.84).
(0.05 MB PDF)

Table S1 *C. cinerea* probe and comparative data. Probe IDs, *C. cinerea* genes, and *S. cerevisiae* and *S. pombe* unique orthologs are indicated for all *C. cinerea* array probes. In addition, *C. cinerea* cluster numbers, genes induced on entry to meiosis in the three species as well as correlation coefficients are shown. As some genes are represented more than once on the array, probes used in comparative analyses are also indicated. In addition, gene names and putative functions of genes in the following categories are listed under separate tabs: MF_co-induced, MF_non_co-induced, NKMF_co-induced, NKMF_co-regulated.
(3.18 MB XLS)

Table S2 GO analysis of *C. cinerea* meiosis-only genes. Gene ontology (GO) enrichment analysis was performed for the 886 genes expressed in meiotic gill tissue only using EASE [39]. Enrichments were discarded if only one gene of a particular class were identified. Enrichments with a Fisher's Exact score >0.01 were also discarded.
(0.05 MB XLS)

Table S3 GO analysis of *C. cinerea* gene clusters. Gene ontology (GO) enrichment analysis was performed for each gene cluster using EASE [39]. Enrichments were discarded if only one gene of a particular class were identified. Enrichments with a Fisher's Exact score >0.01 were also discarded.
(0.08 MB XLS)

Table S4 An expanded inventory of meiotic process genes. Genes are included if they are designated as meiotic function (identified as involved in DNA repair, recombination, replication, or meiosis by gene ontology) and also fulfill one or more of the following criteria: (1) Genes changing significantly during *C. cinerea* meiosis (C) that also have single, unambiguous orthologs in *S. pombe* and *S. cerevisiae* as defined by OrthoMCL (orthologs as indicated), (2) core meiotic genes as defined by [38] (M), (3) genes with characterized *C. cinerea* meiotic functions (as referenced). Mcm2, which is part of the MCM complex but has an FDR>10, and *bad42*, which is known to be critical for meiosis in *C. cinerea*, but for which lack of current known orthologs prevents “meiotic function” designation, are also included. In the indicated pair-wise species comparisons, co-induced genes (+) and genes with a correlation coefficient >0.5 (◊) are shown. For *C. cinerea*, genes with expression only in meiotic tissue are highlighted in bold. Pair-wise comparisons marked “n/a” are those for which comparative data are not available, as gene orthology is not currently apparent in those species; “n/d” indicates a lack of expression data for *C. cinerea*. *C. cinerea has a *hop2* ortholog (CC1G_02025), but no microarray oligonucleotide.
(0.18 MB DOC)

Table S5 qRT-PCR primers. Primers used for amplification of time point cDNA for qRT-PCR.
(0.02 MB XLS)
