# Supplementary figures and images for: Analysis of the Basidiomycete Coprinopsis cinerea Reveals Conservation of the Core Meiotic Expression Program over Half a Billion Years of Evolution
Source: PLoS Genet. 2010 Sep 23;6(9):e1001135. doi: 10.1371/journal.pgen.1001135 (PMC2944786; doi:10.1371/journal.pgen.1001135)

Figure S1

2851 genes

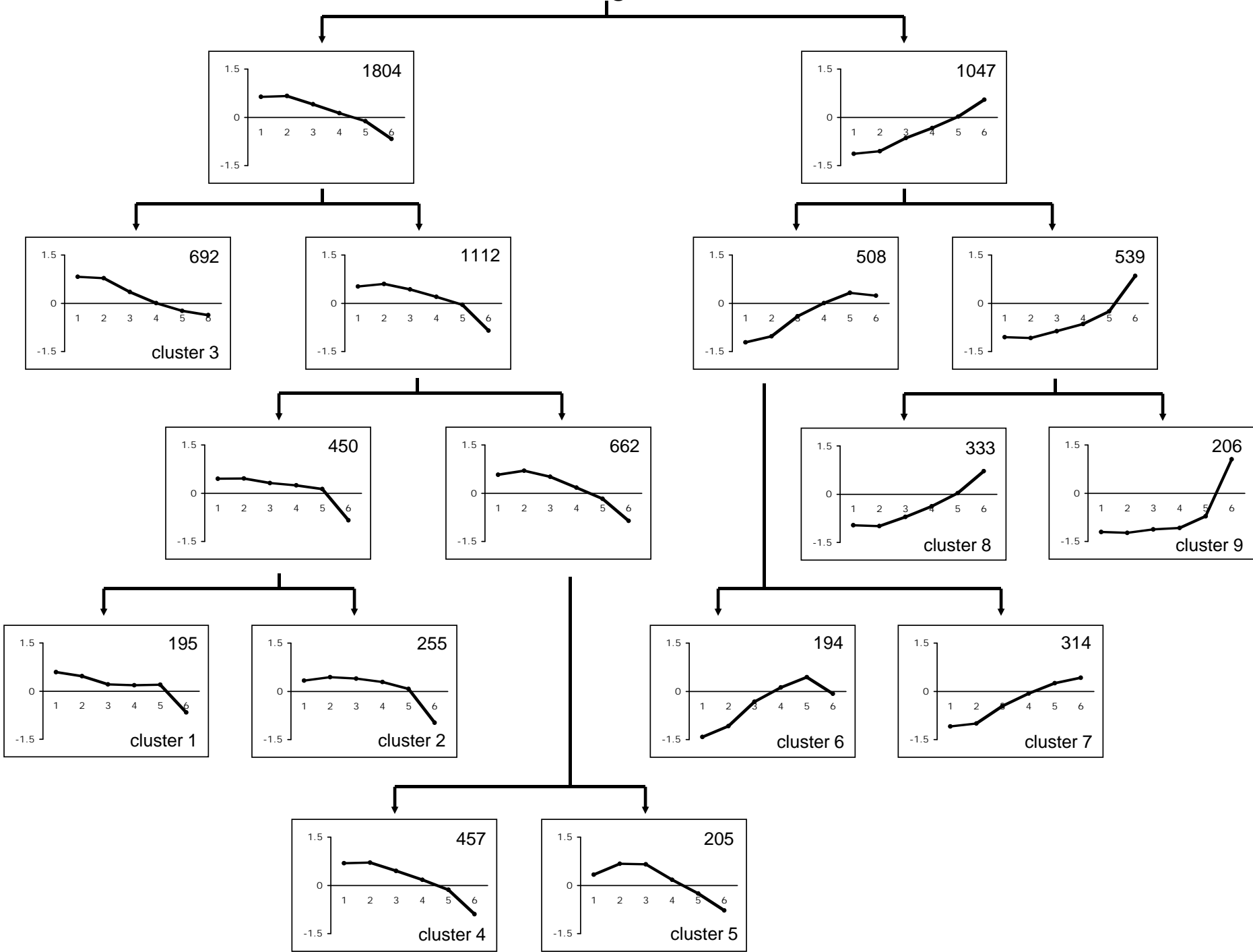

Supplement: Figure S1 — Schematic of gene clustering strategy. The 2,851 probes identified as changing in expression by SAM at an FDR less than 10% were grouped into clusters using a successive bifurcation strategy, as illustrated. (0.02 MB PDF) [file pgen.1001135.s001.pdf]

Figure S2

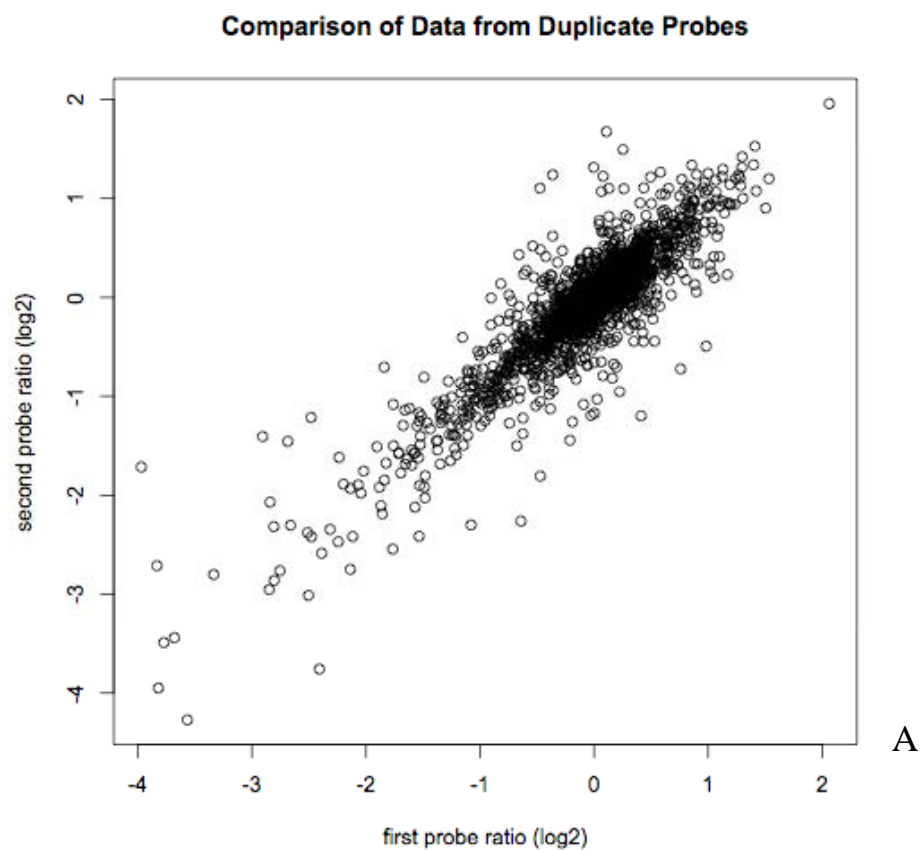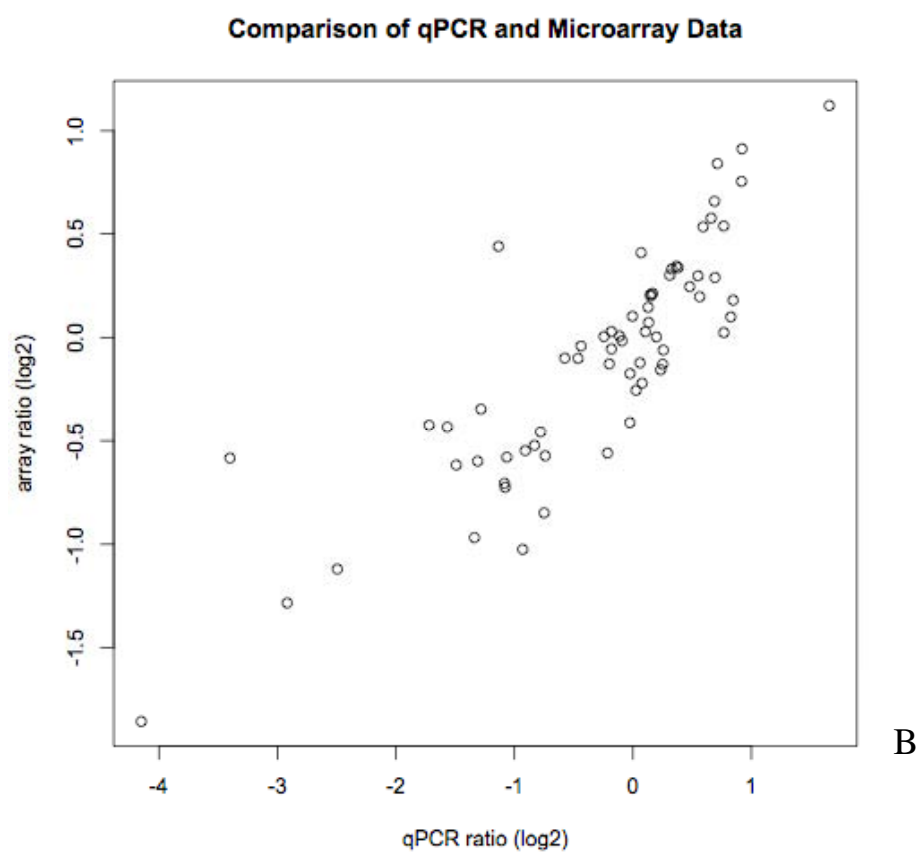

Supplement: Figure S2 — Assessment of microarray probe performance. Array probes were tested for reliability by comparison of expression with a second probe for the same transcript (A; correlation coefficient = 0.89) and by comparison with qPCR expression data (B; correlation coefficient = 0.84). (0.05 MB PDF) [file pgen.1001135.s002.pdf]
