# Supplementary material for: Analysis of the Basidiomycete Coprinopsis cinerea Reveals Conservation of the Core Meiotic Expression Program over Half a Billion Years of Evolution
Source: PLoS Genet. 2010 Sep 23;6(9):e1001135. doi: 10.1371/journal.pgen.1001135 (PMC2944786; doi:10.1371/journal.pgen.1001135)
Supplement: Table S4 — An expanded inventory of meiotic process genes. Genes are included if they are designated as meiotic function (identified as involved in DNA repair, recombination, replication, or meiosis by gene ontology) and also fulfill one or more of the following criteria: (1) Genes changing significantly during C. cinerea meiosis (C) that also have single, unambiguous orthologs in S. pombe and S. cerevisiae as defined by OrthoMCL (orthologs as indicated), (2) core meiotic genes as defined by [38] (M), (3) genes with characterized C. cinerea meiotic functions (as referenced). Mcm2, which is part of the MCM complex but has an FDR>10, and bad42, which is known to be critical for meiosis in C. cinerea, but for which lack of current known orthologs prevents “meiotic function” designation, are also included. In the indicated pair-wise species comparisons, co-induced genes (+) and genes with a correlation coefficient >0.5 (◊) are shown. For C. cinerea, genes with expression only in meiotic tissue are highlighted in bold. Pair-wise comparisons marked “n/a” are those for which comparative data are not available, as gene orthology is not currently apparent in those species; “n/d” indicates a lack of expression data for C. cinerea. *C. cinerea has a hop2 ortholog (CC1G_02025), but no microarray oligonucleotide. (0.18 MB DOC) [file pgen.1001135.s006.doc]

**Table S4: An expanded inventory of “meiotic process” gene**s

| **Scer name (Ccin name)** | **Putative function** | **Ccin/ Spom** | **Ccin/ Scer** | **Scer/**  **Spom** | **Ccin oligo ID** | **Spom gene** | **Scer gene** | **Evidence** |
| --- | --- | --- | --- | --- | --- | --- | --- | --- |
| *ada2* | transcription coactivator | **+** | **+** | **+ ◊** | CC_OL_016_O18 | SPCC24B10.08c | YDR448W | **C** |
| *arp8* | actin-related protein involved in chromatin remodeling |  | **◊** | **◊** | CC_OL_004_H21 | SPAC664.02c | YOR141C | **C** |
| *ask1* | aids in chromosome segregation |  | **◊** | **+** | CC_OL_031_N15 | SPBC27.02c | YKL052C | **C** |
| ***bad42*** | required for meiotic synapsis | n/a | n/a | n/a | CC_OL_004_O04 | - | - | **C**, [64] |
| *bur6* | transcription regulator | **+ ◊** |  |  | CC_OL_003_K01 | SPAC17G8.03c | YER159C | **C** |
| *cdc28* | catalytic subunit of the main cell cycle cyclin-dependent kinase | **+** |  |  | CC_OL_035_P08 | SPBC11B10.09 | YBR160W | **C** |
| *cdc45* | DNA replication initiation factor |  | **+ ◊** |  | CC_OL_024_G02 | SPAC17D4.02 | YLR103C | **C** |
| *cdc8* | essential for meiotic and mitotic DNA replication | **+** | **+ ◊** | **+** | CC_OL_020_K06 | SPCC70.07c | YJR057W | **C** |
| *clf1* | essential spliceosome assembly factor |  | **◊** | **+ ◊** | CC_OL_012_H03 | SPBC31F10.11c | YLR117C | **C** |
| *ctf4* | chromatin associated protein required for sister-chromatid cohesion | **◊** |  |  | CC_OL_022_F02 | SPAPB1E7.02c | YPR135W | **C** |
| ***dmc1*** | involved in pairing and strand exchange to repair double-strand breaks | **+ ◊** | **+** | **+** | CC_OL_004_D22 | SPAC8E11.03c | YER179W | **C**, **M**, [38], [88] |
| *dnl4* (*lig4*) | involved in non-homologous end joining and meiosis | **+** | **+ ◊** | **+** | CC_OL_032_L09 | SPCC1183.05c | YOR005C | **C**, [71] |
| *dyn1* | heavy chain dynein; required for spindle assembly, bouquet formation and chromosome movement | **+** |  |  | CC_OL_008_P05 | SPAC1093.06c | YKR054C | **C** |
| *eaf3* | plays a role in regulating Ty1 transposition |  |  | **◊** | CC_OL_028_I18 | SPAC23H4.12 | YPR023C | **C** |
| *eaf6* | part of acetyltransferase complex that acetylates histone H4 | **+** |  |  | CC_OL_027_B08 | SPAC6F6.09 | YJR082C | **C** |
| *elc1* | transcription elongation |  | **+ ◊** |  | CC_OL_015_G12 | SPBC1861.07 | YPL046C | **C** |
| *glc7* | involved in many processes including glycogen metabolism, sporulation, and mitosis | **◊** | **◊** | **◊** | CC_OL_001_N03 | SPBC776.02c | YER133W | **C** |
| ***hfm1* (*mer3*)** | DNA helicase involved in DSB conversion, crossover control, Holliday junction unwinding | n/a | **+** | n/a | CC_OL_002_D18 | - | YGL251C | **C,** **M**, [88] |
| *hog1* | mediates the recruitment and activation or RNA polymerase |  | **◊** |  | CC_OL_008_O04 | SPAC24B11.06c | YLR113W | **C** |
| ***hop1*** | DNA binding protein required for homologous chromosome synapsis and chiasma formation | **+ ◊** | **+** | **+** | CC_OL_002_F05 | SPBC1718.02 | YIL072W | **C**, **M**, (b) |
| *hop2* | ensures synapsis between homologs, promotes homolog pairing and meiotic DSB repair | n/a | n/a | **+** | * | SPAC222.15 | YGL033W | **M** |
| *hrr25* | subunit of monopolin, which delays anaphase if kinetochores are improperly oriented | **◊** | **+ ◊** | **◊** | CC_OL_020_C16 | SPBC3H7.15 | YPL204W | **C** |
| *irr1* (*scc3*) | subunit of cohesin, which holds sister chromatids together after replication in meiosis and mitosis | **+ ◊** | **+ ◊** | **+** | CC_OL_004_D18 | SPAC17H9.20 | YIL026C | **C**, **M**, (c) |
| *lin1* | interacts with Irr1; may link proteins for chromosome segregation, mRNA splicing and DNA replication | **+** | **+** | **+** | CC_OL_016_O05 | SPBC83.09c | YHR156C | **C** |
| *mad3* | spindle assembly checkpoint subunit | **+** | **+** | **+ ◊** | CC_OL_027_O18 | SPCC1795.01c | YJL013C | **C** |
| *mcm2* | component of the Mcm2-7 complex, involved in replication |  | **+ ◊** |  | CC_OL_017_I05 | SPBC4.04c | YBL023C |  |
| *mcm3* | component of the Mcm2-7 complex, involved in replication |  | **+ ◊** |  | CC_OL_028_E18 | SPCC1682.02c | YEL032W | **C** |
| *mcm4* | component of the Mcm2-7 complex, involved in replication | **◊** | **+** |  | CC_OL_024_O24 | SPCC16A11.17 | YPR019W | **C** |
| *mcm5* | component of the Mcm2-7 complex, involved in replication | **+ ◊** | **+** | **+** | CC_OL_018_M04 | SPAC1B2.05 | YLR274W | **C** |
| *mcm6* | component of the Mcm2-7 complex, involved in replication | **+** | **+ ◊** | **+** | CC_OL_019_K07 | SPBC211.04c | YGL201C | **C** |
| *mcm7* | component of the Mcm2-7 complex, involved in replication | **◊** | **+ ◊** | **◊** | CC_OL_002_F18 | SPBC25D12.03c | YBR202W | **C** |
| *mec1* | genome integrity checkpoint protein, monitors and participates in meiotic recombination |  |  | **◊** | CC_OL_005_B13 | SPBC216.05 | YBR136W | **C** |
| *mgm101* | involved in mitochondrial genome maintenance |  | **◊** |  | CC_OL_021_H20 | SPBC30D10.08 | YJR144W | **C** |
| *mih1* | involved in cell cycle control; regulates the phosphorylation state of Cdc28 | **+ ◊** | **+** | **+** | CC_OL_014_H23 | SPAC24H6.05 | YMR036C | **C** |
| ***mlh1*** | required for mismatch repair and crossing over in meiosis | **+** | **+ ◊** | **+** | CC_OL_014_N23 | SPBC1703.04 | YMR167W | **C**, **M**, (c) |
| *mlh2* | involved in mismatch repair and meiotic recombination; forms a complex with Mlh1 | n/a | n/a | n/a | - | - | YLR035C | **M** |
| *mlh3* | involved in mismatch repair and meiotic recombination; forms a complex with Mlh1 | n/a | n/a | n/a | - | - | YPL164C | **M** |
| *mms21* | SUMO ligase involved in chromosomal organization and DNA repair | **+** | **◊** |  | CC_OL_027_H05 | SPAC16A10.06c | YEL019C | **C** |
| *mnd1* | required for recombination and meiotic nuclear division | **+ ◊** | **+** | **+ ◊** | CC_OL_004_I02 | SPAC13A11.03 | YGL183C | **C**, **M**, (b) |
| *mre11* | part of MRN complex that processes double-strand breaks in meiosis | **+ ◊** | **+ ◊** | **+** | CC_OL_004_E14 | SPAC13C5.07 | YMR224C | **C**, **M**, [8] |
| *msc1* | mutant is defective in directing meiotic recombination events to homologous chromatids |  |  | **+** | CC_OL_028_K01 | SPBC365.12c | YML128C | **C** |
| ***msh1*** | mitochondrial DNA repair | **+ ◊** | **◊** |  | CC_OL_004_P09 | SPAC13F5.01c | YHR120W | **C**, (d) |
| *msh2* | involved in mismatch repair | **◊** | **+ ◊** | **◊** | CC_OL_021_N15 | SPBC19G7.01c | YOL090W | **C**, **M**, (d) |
| *msh4* | involved in meiotic recombination, required for normal levels of crossing over | n/a | n/d | n/a | CC_OL_023_K13 | - | YFL003C | **M**, (d) |
| ***msh5*** | facilitates and marks meiotic crossovers; in *C. cinerea,* required for meiotic DNA replication | n/a | **+** | n/a | CC_OL_004_F16 | - | YDL154W | **C**, **M**, [89] |
| *msh6* | involved in mismatch repair |  | **◊** |  | CC_OL_021_N08 | SPCC285.16c | YDR097C | **C,** **M**, (d) |
| *nam8* | RNA binding protein, required for meiotic recombination, meiosis-specific splicing of MER2 | **◊** | **◊** |  | CC_OL_035_A15 | SPBC23E6.01c | YHR086W | **C** |
| *noc3* | replication initiation | **◊** |  |  | CC_OL_011_F21 | SPBC887.03c | YLR002C | **C** |
| *nse4* | nuclear protein that plays a role in the function of the Smc5p-Rhc18p complex | **◊** | **+ ◊** | **◊** | CC_OL_024_H14 | SPBC20F10.04c | YDL105W | **C** |
| *orc2* | subunit of the origin recognition complex, directs DNA replication | **+** |  | **◊** | CC_OL_032_J19 | SPBC685.09 | YBR060C | **C** |
| ***orc5*** | subunit of the origin recognition complex, directs DNA replication |  | **+ ◊** |  | CC_OL_021_I24 | SPBC646.14c | YNL261W | **C** |
| *paf1* | required for full expression of a subset of cell cycle-regulated genes | **◊** |  | **◊** | CC_OL_013_P05 | SPAC664.03 | YBR279W | **C** |
| ***pds5*** | establishment and maintenance of sister chromatid condensation and cohesion, colocalizes with cohesin | **+** | **◊** | **◊** | CC_OL_004_D09 | SPAC110.02 | YMR076C | **M** |
| ***pms1*** | mismatch repair in mitosis and meiosis; functions as a heterodimer with Mlh1 | **+** | **+ ◊** | **+** | CC_OL_003_G22 | SPAC19G12.02c | YNL082W | **M** |
| *pol5* | DNA polymerase |  |  | **◊** | CC_OL_007_M14 | SPBC14C8.14c | YEL055C | **C** |
| *pri2* | subunit of DNA primase, required for DNA synthesis and double-strand break repair | **+ ◊** | **◊** | **◊** | CC_OL_031_D15 | SPBC17D11.06 | YKL045W | **C** |
| ***psf3*** | assembly of replication machinery |  | **+** |  | CC_OL_009_J20 | SPAC227.16c | YOL146W | **C** |
| ***rad1*** | cleaves single-stranded DNA during nucleotide excision repair and double-strand break repair | **+** | **+** | **+** | CC_OL_004_D10 | SPCC970.01 | YPL022W | **M** |
| *rad18* | involved in postreplication repair | **◊** |  |  | CC_OL_010_F20 | SPBC1734.06 | YCR066W | **M** |
| *mcd1*  (*rad21.1*) | subunit of cohesin, which holds sister chromatids together after replication in meiosis and mitosis | **+** |  | **◊** | CC_OL_004_F17 | SPCC33817.c | YDL003W | **C**, **M**, (e) |
| *mcd1*  (*rad21.2*) | subunit of cohesin, which holds sister chromatids together after replication in meiosis and mitosis | **+ ◊** | **◊** | **◊** | CC_OL_004_O14 | SPCC33817.c | YDL003W | **C**, **M**, (e) |
| *rad23* | recognizes and binds damaged DNA during nucleotide excision repair |  | **+** | **◊** | CC_OL_001_J10 | SPBC2D10.12 | YEL037C | **C** |
| *rad27* (*fen1*) | flap endonuclease | **+** | **+ ◊** | **+** | CC_OL_028_G20 | SPAC3G6.06c | YKL113C | **C**, [73] |
| *rad50* | part of MRN complex that processes double-strand breaks in meiosis | **+ ◊** | **+ ◊** | **+ ◊** | CC_OL_004_O16 | SPAC1556.01c | YNL250W | **C**, **M**, [10, 54, 90] |
| *rad51* | involved in strand exchange to repair double-strand breaks | **+ ◊** | **+** | **+** | CC_OL_001_E13 | SPAC644.14c | YER095W | **M**, [12,91] |
| *rad52* | stimulates strand exchange by facilitating Rad51 binding; involved in repair of double-strand breaks | **+** | **+ ◊** | **+** | CC_OL_004_F20 | SPAC30D11.10 | YML032C | **M** |
| *rad54* | stimulates strand exchange by modifying topology of dsDNA; involved in repair of double-strand breaks | **+** | **+** | **+** | CC_OL_004_P08 | SPAC15A10.03c | YGL163C | **C** |
| ***rec8*** | meiosis-specific subunit of cohesin | **+ ◊** | **+** | **+** | CC_OL_004_P24 | SPBC29A10.14 | YPR007C | **C**, **M**, (e) |
| *rfa1* | subunit of Replication Protein A (RPA), involved in DNA replication, repair, and recombination | **+** | **+ ◊** | **+** | CC_OL_002_D15 | SPBC660.13c | YAR007C | **C** |
| *rfa2* | subunit of Replication Protein A (RPA), involved in DNA replication, repair, and recombination | **◊** | **+ ◊** |  | CC_OL_006_D14 | SPCC1753.01c | YNL312W | **C** |
| *rfc3* | subunit of replication factor C | **◊** | **+ ◊** | **◊** | CC_OL_008_G04 | SPAC27E2.10c | YNL290W | **C** |
| *rfc4* | subunit of replication factor C |  | **+ ◊** | **◊** | CC_OL_029_P07 | SPAC1687.03c | YOL094C | **C** |
| *rfc5* | subunit of replication factor C |  | **+ ◊** |  | CC_OL_033_A10 | SPBC83.14c | YBR087W | **C** |
| *rpb9* | contacts DNA; mutations affect transcription start site; involved in telomere maintenance |  |  |  | CC_OL_017_L17 | SPAPYUG7.04c | YGL070C | **C** |
| *rpd3* | regulates transcription and silencing | **◊** | **+ ◊** | **◊** | CC_OL_001_E02 | SPBC36.05c | YNL330C | **C** |
| *rsc8* | component of the RSC chromatin remodeling complex; essential for viability and mitotic growth |  | **◊** |  | CC_OL_033_C06 | SPAC23H3.10 | YFR037C | **C** |
| *sba1* | co-chaperone that binds to and regulates Hsp90 family chaperones,can regulate telomerase activity |  | **◊** |  | CC_OL_020_B15 | SPAC9E9.13 | YKL117W | **C** |
| *scc2* (*rad9*) | involved in cohesin loading, chromosome condensation, and synapsis | **◊** | **+ ◊** | **◊** | CC_OL_004_F15 | SPAC31A2.05c | YDR180W | **C**, (f) |
| ***sfh1*** | component of the RSC chromatin remodeling complex; required for cell cycle progression | **+** | **+ ◊** | **+** | CC_OL_031_D11 | SPCC16A11.14 | YLR321C | **C** |
| *ski8* | involved in double-strand break formation during meiotic recombination | **+** | **◊** | **◊** | CC_OL_030_A09 | SPBC32F12.02 | YGL213C | **C** |
| *sld3* | involved in the initiation of DNA replication |  | **◊** | **+** | CC_OL_025_D14 | SPAC24H6.06 | YGL113W | **C** |
| *smc1* | subunit of cohesin, which holds sister chromatids together after replication in meiosis and mitosis | **+ ◊** | **◊** |  | CC_OL_013_N18 | SPBC29A10.04 | YFL008W | **C**, **M**, [92] |
| *smc3* | subunit of cohesin, which holds sister chromatids together after replication in meiosis and mitosis | **+ ◊** | **◊** |  | CC_OL_004_P13 | SPAC10F6.09c | YJL074C | **C**, **M**, (c) |
| *smc5* | Structural Maintenance of Chromosomes (SMC) protein; required for growth and DNA repair |  | **+ ◊** |  | CC_OL_006_I16 | SPAC14C4.02c | YOL034W | **M** |
| ***spo11*** | forms double-strand breaks in meiosis | **+ ◊** | **+** | **+** | CC_OL_004_E17 | SPAC17A5.11 | YHL022C | **C**, **M**, [7, 10, 93] |
| *spo7* | required for normal nuclear envelope morphology and sporulation | **◊** |  |  | CC_OL_032_N05 | SPBC902.03 | YAL009W | **C** |
| ***srs2*** | DNA repair and co-ordination of meiosis | **+ ◊** | **+** | **+** | CC_OL_024_J12 | SPAC4H3.05 | YJL092W | **C** |
| *sub2* | required for nuclear mRNA export; RNA helicase involved in splicing | **◊** |  | **◊** | CC_OL_019_L24 | SPAC17G6.14c | YDL084W | **C** |
| *swe1* | protein kinase that regulates the G2/M transition by inhibition of Cdc28p kinase activity |  |  | **+** | CC_OL_001_I18 | SPCC18B5.03 | YJL187C | **C** |
| *tel1* | protein kinase involved in telomere length regulation and DNA damage cell cycle checkpoint | **+ ◊** |  |  | CC_OL_004_P14 | SPCC23B6.03c | YBL088C | **C** |
| *tho2* | required for efficient transcription elongation and involved in elongation-associated recombination | **+** | **+ ◊** | **+** | CC_OL_002_P11 | SPAC1D4.14 | YNL139C | **C** |
| *tif34* | subunit of the core complex of translation initiation factor 3 |  |  |  | CC_OL_020_J15 | SPAC4D7.05 | YMR146C | **C** |
| *ulp2* | role in chromatid cohesion and recovery from checkpoint arrest | **+ ◊** | **+** | **+** | CC_OL_004_J21 | SPAC17A5.07c | YIL031W | **C** |
| *urn1* | role in chromosome cohesion, recovery from DNA damage/replication checkpoint arrest | **◊** | **+** |  | CC_OL_019_B18 | SPAC13C5.02 | YPR152C | **C** |
| *vam7* | component of the vacuole SNARE complex involved in vacuolar morphogenesis |  | **◊** |  | CC_OL_020_B01 | SPCC594.06c | YGL212W | **C** |
| *vps75* | proposed role in vacuolar protein sorting and in double-strand break repair |  | **◊** | **◊** | CC_OL_009_G10 | SPBC36B7.08c | YNL246W | **C** |
| *xrs2* (*nbs1*) | part of MRN complex that processes double-strand breaks in meiosis | **+** | **+** | **+ ◊** | CC_OL_003_L19 | SPBC6B1.09c | YDR369C | (g) |
| *yaf9* | may function to antagonize silencing near telomeres | **◊** |  |  | CC_OL_019_J06 | SPAC17G8.07 | YNL107W | **C** |
| *yra1* | nuclear protein that binds to RNA required for export of poly(A)+ mRNA from the nucleus |  | **+** | **◊** | CC_OL_001_M21 | SPBC1D7.04 | YDR381W | **C** |

**Additional references not cited in the main text**

88. Ishii S, Koshiyama A, Hamada FN, Nara TY, Iwabata K, et al. (2008) Interaction between Lim15/Dmc1 and the homologue of the large subunit of CAF-1: a molecular link between recombination and chromatin assembly during meiosis. FEBS J 275: 2032-2041.

89. Cummings WJ, Merino ST, Young KG, Li LB, Johnson CW, et al. (2002) The *Coprinus cinereus* adherin Rad9 functions in Mre11-dependent DNA repair, meiotic sister-chromatid cohesion, and meiotic homolog pairing. Proc Natl Acad Sci U S A 99: 14958-14963.

90. Many AM, Melki CS, Savytskyy OP, Maillet DS, Acharya SN, et al. (2009) Meiotic localization of Mre11 and Rad50 in wild type, *spo11-1*, and MRN complex mutants of *Coprinus cinereus*. Chromosoma 118: 471-486.

91. Nara T, Hamada F, Namekawa S, Sakaguchi K (2001) Strand exchange reaction in vitro and DNA-dependent ATPase activity of recombinant Lim15/Dmc1 and Rad51 proteins from *Coprinus cinereus*. Biochemical and Biophysical Research Communications 285: 92-97.

92. Muraguchi H, Abe K, Nakagawa M, Nakamura K, Yanagi SO (2008) Identification and characterisation of structural maintenance of chromosome 1 (*smc1*) mutants of *Coprinopsis cinerea*. Molecular Genetics and Genomics 280: 223-232.

93. Cummings WJ, Celerin M, Crodian J, Brunick LK, Zolan ME (1999) Insertional mutagenesis in *Coprinus cinereus*: use of a dominant selectable marker to generate tagged, sporulation-defective mutants. Current Genetics 36: 371-382.
